# Invasive shrub re-establishment following management has contrasting effects on biodiversity

**DOI:** 10.1038/s41598-019-40654-y

**Published:** 2019-03-11

**Authors:** Luke S O’Loughlin, Ben Gooden, Claire N. Foster, Christopher I. MacGregor, Jane A. Catford, David B. Lindenmayer

**Affiliations:** 1 Fenner School of Environment and Society, The Australian National University, Canberra, ACT 2601 Australia; 2 School of Environmental Science, Charles Sturt University, Albury, NSW 2640 Australia; 3 Centre for Sustainable Ecosystem Solutions, School of Biological Sciences, University of Wollongong, Wollongong, NSW 2522 Australia; 4 CSIRO Health and Biosecurity, GPO Box 1700, Canberra, ACT 2601 Australia; 5 Threatened Species Recovery Hub of the National Environment Science Programme, Fenner School of Environment and Society, The Australian National University, Canberra, ACT 2601 Australia; 6 Biological Sciences, University of Southampton, Southampton, SO17 1BJ UK; 7 Department of Geography, King’s College London, London, WC2B 4BG UK

## Abstract

Effective control of an invasive species is frequently used to infer positive outcomes for the broader ecosystem. In many situations, whether the removal of an invasive plant is of net benefit to biodiversity is poorly assessed. We undertook a 10-year study on the effects of invasive shrub management (bitou bush, *Chrysanthemoides monilifera* ssp. *rotundata*) on native flora and fauna in a eucalypt forest in south-eastern Australia. Bitou bush eradication is a management priority, yet the optimal control regime (combination of herbicide spray and fire) is difficult to implement, meaning managed sites have complex management histories that vary in effectiveness of control. Here we test the long-term response of common biodiversity indicators (species richness, abundance and diversity of native plants, birds, herpetofauna and small mammals) to both the management, and the post-management status of bitou bush (% cover). While average bitou bush cover decreased with management, bitou bush consistently occurred at around half of our managed sites despite control efforts. The relationship between biodiversity and bitou bush cover following management differed from positive, neutral or negative among species groups and indicators. Native plant cover was lower under higher levels of bitou bush cover, but the abundance of birds and small mammals were positively related to bitou bush cover. Evidence suggests that the successful control of an invader may not necessarily result in beneficial outcomes for all components of biodiversity.

## Introduction

Invasive plants significantly threaten biodiversity and ecosystem function^1,2^, but limited conservation resources mean that only a small proportion of them are managed^3^. Ideally, invasive species that have the greatest impacts on the recipient ecosystem would be prioritised for ecological management^4^. However, ecological impacts are difficult to quantify^1,5^, meaning in many situations, invasive plants that have large ranges and reach high abundance tend to be targeted for management without robust quantitative assessments of impact^6,7^. This precautionary approach is based on the established correlative relationship between these measures of a species ‘invasiveness’ and its ecological impact^8,9^, but does not capture the strong context-dependency and variability (in both magnitude and direction) intrinsic of invasive plant impacts^1,6,10^. Unlike other applications of the ecological surrogacy concept^11^, the accuracy, stability and certainty of the relationship between invasiveness and impact is rarely evaluated before it is used to inform management.

It is also difficult to quantify whether management that aims to reduce the spread and abundance of an invasive plant also delivers positive ecosystem outcomes. As a consequence, the effective management of invasive plants is typically assumed to reflect benefits for native biodiversity. Most studies of invasive plant management only monitor the response of the invader to management, not the responses of native species^8,12^. In fact, when ecosystem recovery *is* measured following management intervention, negative and mixed outcomes are common^13^, the establishment of other invaders is frequently observed^14^, and sites cleared of invasive plants may show no evidence of returning to the historical native community even decades later^15^. Similarly, failure to consider the impacts of invasive plant invasion or management on non-plant taxa^1,10^ risks a misallocation of resources and/or potentially detrimental ecosystem outcomes for threatened species^12,16,17^. Evaluating how well the response of the invader represents the response of biodiversity to management intervention is critical to informing evidence-based management.

Here, we sought to test the assumption that invasive species’ responses to management indicate responses of native biodiversity to that management. We tested this assumption in a forest ecosystem invaded by an alien shrub – bitou bush (*Chrysanthemoides monilifera* ssp. *rotundata*) – in south-eastern Australia. Negative ecological effects of bitou bush invasion, and positive effects of successful bitou bush management, on native plant diversity have been widely documented (see^18^). However, neutral or positive responses of plants and animals to bitou bush invasion, and negative effects of bitou bush management, also have been observed, leading most studies to conclude that the impacts of bitou bush should not be generalised across time, space or native species or ecosystems^19,20^. Therefore, context-specific evaluation is needed to adequately capture the variability in impacts of both bitou bush invasion (establishment of the invader) and management (actions to reduce/remove the invader). It is well-established that bitou bush creates an understorey that is more structurally dense and shaded than native vegetation^20^, with altered litter and nitrogen-cycling dynamics^21^. However, it is less established whether, over the long-term, these ecosystem changes have consistently negative effects, and whether the removal of bitou bush has consistently positive outcomes for biodiversity, particularly for native fauna.

In this 10-year study, we quantified the response of native plants, birds, herpetofauna (reptiles and amphibians) and small mammals to the long-term effects of ongoing bitou bush management. While previous studies have focused on the short-term effects of management within this program^22,23^, the longer-term cumulative effects of ongoing management, combined with the impacts of invader re-establishment (defined as the percentage live foliage cover of bitou bush at various points in time following management), remain largely unexplored. In a system where management does not result in eradication, and the re-establishment of bitou bush in managed sites is variable, we ask three key questions: (1) What are the properties of ongoing management (i.e. frequency of, and time since last, fire or spray) that best explain the decline or re-establishment of bitou bush? (2) Does the re-establishment of bitou bush following management have a significant negative effect on the richness, abundance and diversity of different biotic groups? and (3) Are the properties of on-going bitou bush management also having predictable effects on the richness, abundance and diversity of different biotic groups?

## Results

A total of 174 vascular plant, 108 bird, 11 reptile, 8 amphibian and 13 mammal species were recorded during this study (see Table S1). Introduced species other than bitou bush were rarely observed and included only nine plant species (of which *Hydrocotyle bonariensis* was most common) and two mammal species (black rat *Rattus rattus* and European rabbit *Oryctolagus cuniculus*). Most plant species (88%) were rare, recorded in less than 10% of total quadrats. Yellow-faced honeyeater *Caligavis chrysops*, rainbow lorikeet *Trichoglossus moluccanus* and grey fantail *Rhipidura albiscapa* were the most commonly observed birds, accounting for a total of 25% of all birds recorded. The herpetofauna community was dominated by delicate skink *Lampropholis delicata* and small-eyed snake *Cryptophis nigrescens*, representing 64% and 20% of all observations respectively. Similarly, 82% of all trapped mammals were from two species; brown antechinus *Antechinus stuartii* (48%) and bush rat *Rattus fuscipes* (34%).

### Bitou bush response to management

**Figure 1:**
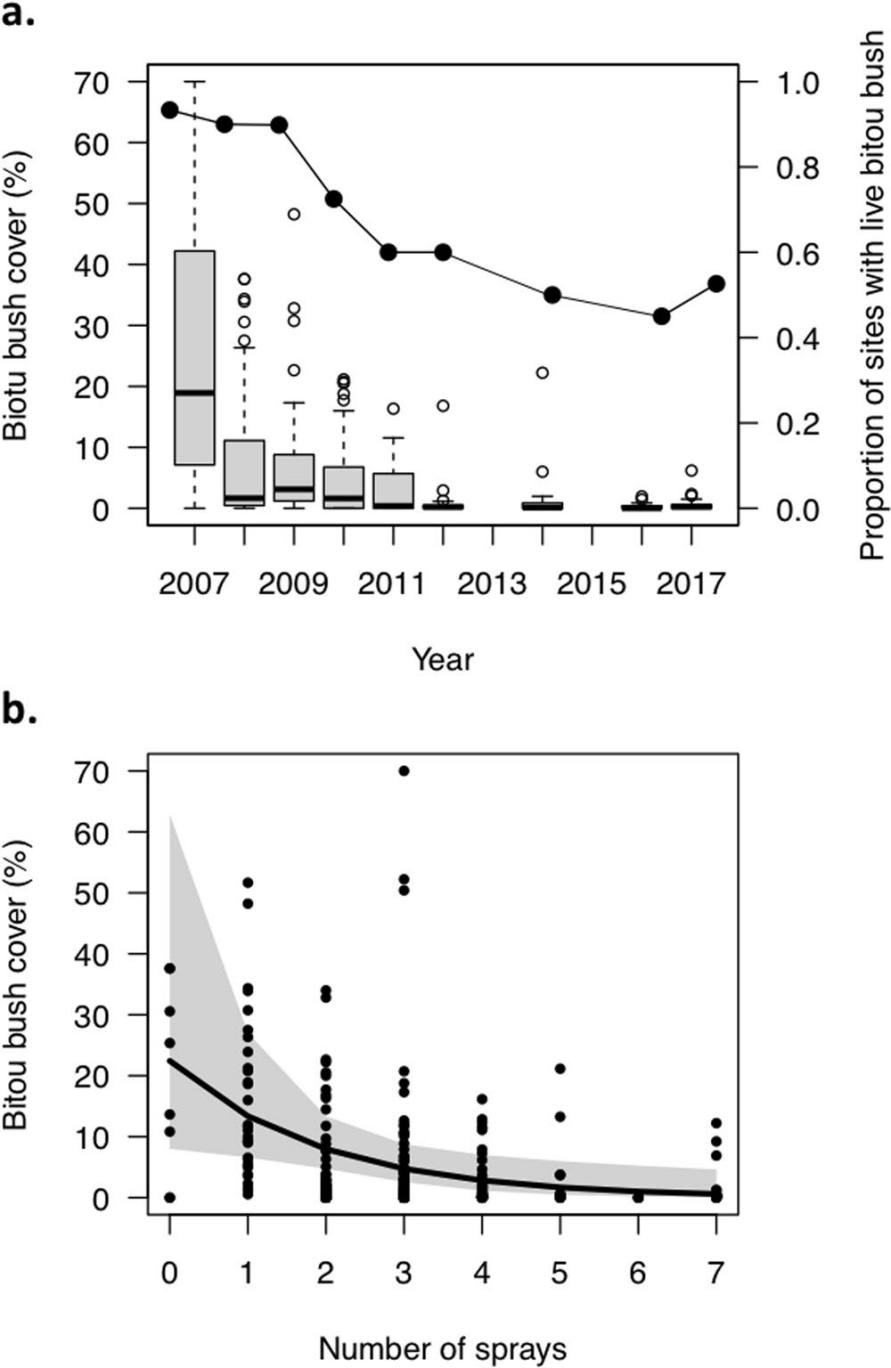
Summary of (**a**) temporal changes in live bitou bush cover post-management and the proportion of managed sites (n = 20) where live bitou bush was still present during the survey period (2007–2017), and (**b**) the relationship between number of glyphosate sprays and the cover of live bitou bush. Predicted values from the top-ranked GLMM and 95% confidence intervals, as well as raw values (closed circles) are shown.

The recommended management for bitou bush comprises spraying with herbicide, burning, then re-spraying. In our study, sites where bitou bush was managed were subject to, on average, 2.15 ± 1.18 SD fires (range = 0 to 4, from 1979) and 4.40 ± 1.67 SD herbicide sprays (range = 2 to 7) between 1997 and early 2017. Sites were monitored while management was (and remains) on-going, with any observation being an average of 2.32 ± 2.30 SD years since either a fire or spray (range = 0 to 10). Average bitou bush cover at managed sites decreased over time, and rarely exceeded 20% cover from 2010 (Fig. 1a). However, the proportion of sites that contained some live bitou bush did not change from 2010, persisting at around 50%, despite management (Fig. 1a). The most strongly-supported model for explaining the cover of bitou bush in managed sites did not include fire frequency, but did include the number of herbicide sprays, which was a consistent factor across all supported models (Table S2A). The cover of live bitou bush was negatively associated with increasing number of sprays (Coeff. = −0.82, 95% CI [−1.51, −0.21], Fig. 1b) irrespective of how fire was used.

### Biodiversity responses to bitou bush re-establishment

**Figure 2:**
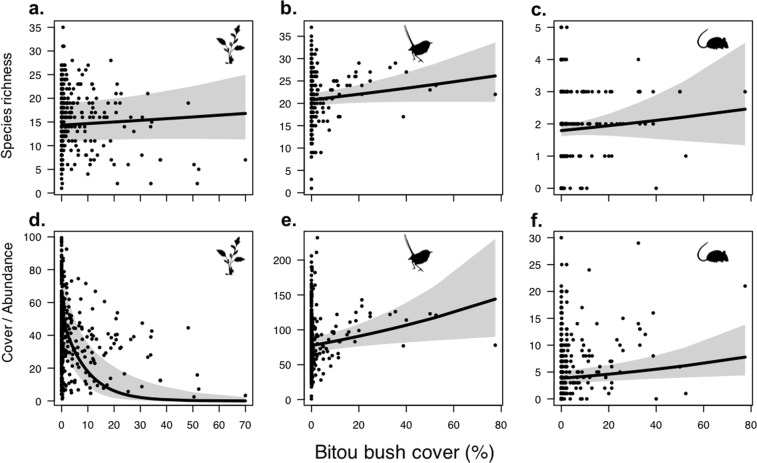
Relationship between live bitou bush cover (%) and site species richness and cover/abundance of (**a**,**d**) plants, (**b**,**e**) birds, and (**c**,**f**) small mammals. Predicted values from the top-ranked GLMM and 95% confidence intervals, as well as raw values (closed circles) are shown. Predictions were made by holding other variables included in the top-ranked model at its mean value (see Table 2). Species richness for each assemblage was calculated as the number of species detected per survey effort for each survey period at each site. Plant cover was calculated as the average percent cover of native species from four 1 × 1 m^2^ quadrats for each survey period for each site. Abundance of fauna was calculated as the number of individuals detected per survey effort for each survey period at each site (see Methods for details). Vector images are courtesy of the Integration and Application Network, University of Maryland Center for Environmental Science (www.ian.umces.edu/symbols/).

As bitou bush was observed to re-establish following management, we tested biodiversity responses to bitou bush cover and properties of ongoing management (i.e. frequency of, and time since last, fire or spray) within the same model. The cover of live bitou bush was included in the top-ranked or a supported model (ΔAICc < 2) to explain the species richness and abundance of native plants, birds, and small mammals (Table S2B–I). However, the species richness of these groups was not significantly associated with bitou bush cover (Table 1, Fig. 2a–c). Instead, increased bitou bush cover was strongly associated with decreased native plant cover, which was found to be close to absent where bitou bush cover exceeded 40% (Table 1, Fig. 2d). There were also weak positive associations between bitou bush cover and the abundance of birds and small mammals, in both cases representing a close to 1-fold increase in abundance where bitou bush cover was highest compared to where it was low (Table 1, Fig. 2e,f), There was no significant relationship between bitou bush cover and herpetofauna abundance (Table 1). Bitou bush cover was not included in any supported models to explain native plant and bird diversity (Table S2J,K), and was unrelated to small mammal diversity (Table 1) despite featuring in a supported model (Table S2L).

**Table 1:** Results of generalised linear mixed models testing the association of live bitou bush cover, fire frequency, time since last spray and any interaction on the species richness, abundance and diversity (Shannon’s Diversity Index) of plant, bird, herpetofauna, and small mammal assemblages.

Indicator	Fixed-effect	Plant		Bird		Herpetofauna		Small mammal	
		Est. (s.e.)	*Z*	Est. (s.e.)	*Z*	Est. (s.e.)	*Z*	Est. (s.e.)	*Z*
Richness	intercept	2.67 (0.14)	**18**.**93**	3.04 (0.04)	**84**.**48**	−0.11 (0.12)	−0.98	0.60 (0.05)	**11**.**17**
	Bitou bush cover	0.02 (0.02)	1.04	0.02 (0.01)	1.81			0.03 (0.03)	0.10
	Fire frequency	0.14 (0.05)	**2**.**53**			0.11 (0.06)	1.78	−0.05 (0.04)	−1.25
	Bitou bush cover x Fire frequency	0.03 (0.02)	1.59						
Abundance	intercept	−1.07 (0.24)	**−4**.**40**	4.38 (0.07)	**61**.**06**	0.45 (0.26)	1.75	1.37 (0.15)	**8**.**88**
	Bitou bush cover	−0.94 (0.23)	**−3**.**30**	0.06 (0.02)	**2**.**58**	0.03 (0.05)	0.70	0.08 (0.03)	**2**.**67**
	Fire frequency	0.68 (0.23)	**2**.**95**					−0.23 (0.08)	**−3**.**03**
	Time since last spray	0.60 (0.15)	**3**.**90**					−0.01 (0.06)	−0.34
Diversity	intercept	2.08 (0.12)	**16**.**89**					1.36 (0.06)	**21**.**86**
	Bitou bush cover							0.01 (0.03)	0.15
	Fire frequency	0.18 (0.05)	**3**.**97**						
	Time since last spray							0.01 (0.04)	0.37

Standardised regression coefficients with standard error (Est. (s.e.)) and Z-values are shown for explanatory variables where they feature in the top-ranked model (or supported model that included BB) for each response variable (see Table S1). Bolded Z-values denote significant effects (*P* < 0.05).

### Biodiversity responses to bitou bush management

**Figure 3:**
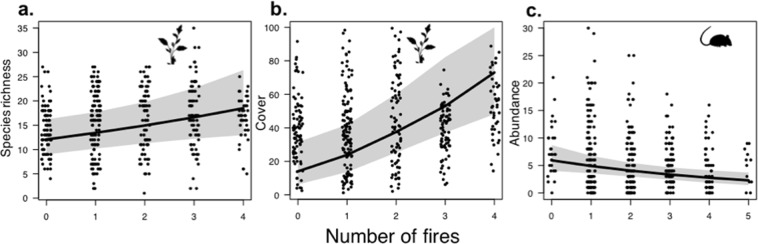
Relationship between fire frequency and (**a**) plant species richness (**b**) native plant cover (%), and (**c**) small mammal abundance. Predicted values from the top-ranked GLMM and 95% confidence intervals, as well as raw values (closed circles, jittered to minimize overlap) are shown. Predictions were made by holding other variables included in the top-ranked model at their mean values (see Table 2). Vector images are courtesy of the Integration and Application Network, University of Maryland Center for Environmental Science (www.ian.umces.edu/symbols/).

Fire frequency was included in the top-ranked models for plant species richness, cover and diversity, herpetofauna species richness, and the species richness and abundance of small mammals. All native plant indicators were positively associated with fire frequency (Table 1, Fig. 3a,b), while increased number of fires had a negative effect on the abundance of small mammals (Table 1, Fig. 3c). Native plant cover was positively associated with time since last spray (Table 1). None of the management variables were significantly associated with the richness or abundance of birds or herpetofauna (see Table 1).

## Discussion

**Figure 4:**
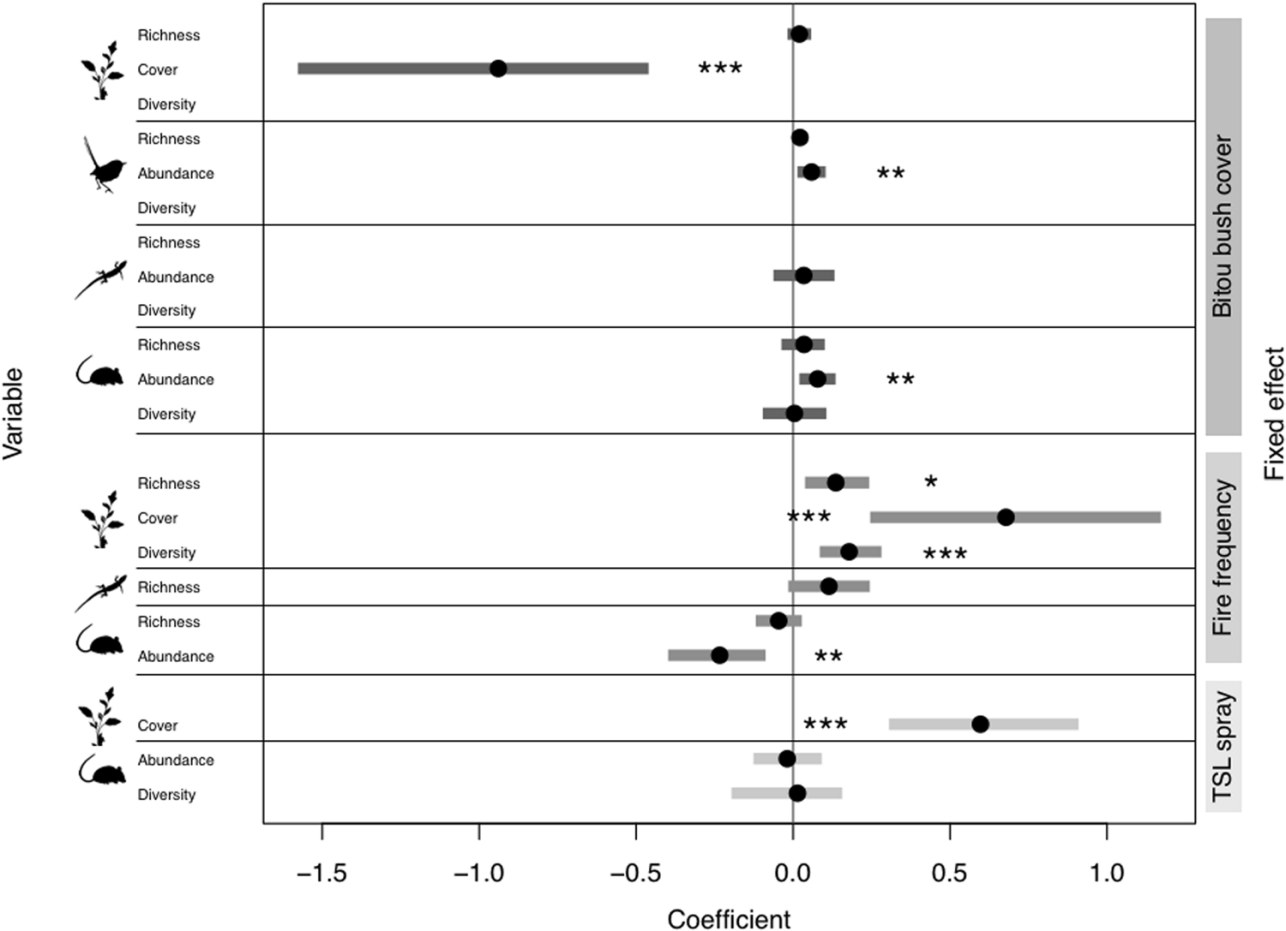
Results of generalised linear mixed models testing the main effects of bitou bush cover, fire frequency, and time since last (TSL) spray on the 12 biodiversity indicators (variables) considered in this study. Estimated coefficients and 95% confidence intervals for the overall effect of live bitou bush cover (%) on the species richness, cover/abundance, and diversity (Shannon’s Diversity Index) of plants, birds, herpetofauna and small mammals. Empty rows indicate where bitou bush cover was not in a supported model for the response of that variable. Management effects (Fire frequency and TSL spray) shown only where they featured in a supported model of a response variable (see Table 2). Confidence intervals of the estimated model coefficient that do not overlap zero indicate a significant association between that indicator and main effect. Stars denote significant effects of *P < 0.05, **P < 0.01 and ***P < 0.001. Vector images are courtesy of the Integration and Application Network, University of Maryland Center for Environmental Science (www.ian.umces.edu/symbols/).

In this 10 year study of invasive shrub management and native biodiversity responses, we found that the extent of invader re-establishment following ongoing management was significantly associated with the abundance of native plants, birds and small mammals, but not the species richness or diversity of those groups (Fig. 4). This association between invader cover and biodiversity indicators supports the intuitive – yet rarely tested – assumption that the response of the invader to management can be used to make some inferences about effects of that management on broader biodiversity patterns. However, the direction and magnitude of those effects differed among biotic groups, with bitou bush re-establishment having a strong negative effect on native plant cover, but a weak positive effect on the abundance of birds and small mammals. These results suggest that reductions in invasive species abundance alone are a poor indicator of positive outcomes for biodiversity as the re-establishment of bitou bush following management was either not important, or was of some small benefit to native fauna (Fig. 4). Instead, determining management efficacy with regards to biodiversity outcomes requires more direct indicators to more accurately infer management has an overall beneficial effect on the managed ecosystem.

Management of bitou bush over the last 10 years has largely been successful, with cover of live bitou bush at managed sites rarely exceeding 20% since 2010 (Fig. 1a). This is encouraging given that the ideal sequence of management (spray-fire-spray) has rarely been achieved^22^. While we found that bitou bush cover at managed sites was low, live bitou bush still persisted at around half of our sites. This represents a significant re-establishment risk given the capacity of bitou bush to spread rapidly and dominate communities^24^. A lack of follow-up management would likely lead to proliferation of bitou bush across the study region. Our monitoring is consistent with the idea that eradication of long-established invasive plants is largely unfeasible^25,26^, and that management should instead aim to control these species below identified impact thresholds, and prevent further spread^5,27^.

Bitou bush re-establishment following management was negatively associated native plant cover. This relationship was expected given the well-documented pattern of native vegetation replacement by successful invasive plants^1,10^, and the capacity of bitou bush to form dense monocultures across its invaded range^20,24^. However, bitou bush re-establishment did not have a similar strongly negative relationship with native plant species richness. These results are similar to other studies that have found eucalypt forest that was heavily invaded by bitou bush still supported a diverse above-ground native plant community, not dissimilar in species richness from uninvaded sites^20,28,29^. However, in other communities, such as fore dune scrublands, bitou bush invasion is strongly associated with significant decreases in native species richness^18,20^. Here, we too found that while native plant species richness may not be strongly responsive to the effectiveness of bitou bush management, the cover of native species is highly contingent on the successful reduction of bitou bush cover.

In contrast to the negative response of native plant cover to bitou bush re-establishment, we found that the abundance of both birds and small mammals increased with higher levels of bitou bush cover following management. This is consistent with our understanding that both these faunal groups benefit from structurally complex mid- and understorey vegetation strata, likely due to the increased resources and protection from predators afforded in denser vegetation^30^. Invasive plants can often act as important habitat for native species^17,31,32^. For example, native birds can be positively associated with dense thickets of invasive blackberry (*Rubus fruticosus*), particularly in highly-modified ecosystems with little residual native vegetation cover (e.g.^33^). Similarly, small mammals are generally more abundant in areas with dense native shrub cover^30^. Our results suggest bitou bush may be functionally analogous to native shrubs from the perspective of small mammals at our sites. Previous research on the effect of bitou bush management found a positive effect of increased cover of dead bitou only for bush rat (*Rattus fuscipes*) abundance, inferring a positive effect of recent spray application^23^. Here, we have identified a previously overlooked positive impact of bitou bush re-establishment on a faunal community thought to be largely unaffected by either the invader or its management. Understanding whether, given sufficient time, native plants can provide the structural complexity and vegetation density that promotes increased animal abundance, thereby providing that small ecological benefit to some fauna that bitou bush currently provides, is an important question for future research. While these benefits of bitou bush re-establishment for fauna were weak compared the negative effect on plant cover, they may become more important in the future, particularly as small mammals have decreased across the National Park in recent years^34^.

We found that herbicide spray frequency was the only important component of the ongoing management that predicted the response of bitou bush. Live bitou bush cover was negatively associated with increased number of glyphosate sprays, regardless of how fire was applied at those sites. Fire is used prior to a spray with the aim of stimulating the germination of fire-cued bitou bush seed^35^, thereby depleting the soil seed bank. However, for logistical reasons, this more regularly does not happen at the ideal time or at all^22^. The lack of a significant fire effect in our study may simply have arisen because the ideal spray-fire-spray sequence was applied too infrequently to allow detection of individual effects of fire (but see^22^) rather than fire not being important in bitou bush control. Bitou bush seed is highly-transient (<1 year longevity^36^) and abundance in the soil seed bank is significantly lower in sparsely-invaded sites compared to heavily invaded sites^19^, indicating that the benefits of burning may be limited at low bitou bush densities. While the ideal management sequence is critical to managing large infestations of bitou bush in the short-term^22^, over the long-term, effective control of small infestations will be contingent upon continued herbicide application, or the integration of other approaches (e.g. hand-pulling emergent seedlings or release of biological control agents) that have been used to control bitou bush elsewhere across its invaded range^24,37^.

We could not directly test the response of biodiversity to spray frequency because it was highly correlated live bitou bush cover. However, we did find native plant cover was positively associated with increased time since spray. This may indicate vegetation recovery from either the spray itself or release from the impacts of bitou bush. Glyphosate is used widely in weed management because of its ready availability and low-cost, despite its varying effectiveness and many non-target impacts^8^. However, the effectiveness and impact of glyphosate is typically assessed only in the short-term. While the application of a general herbicide is known to have immediate negative effects on native plants both in our study system and elsewhere^8,22^, we found that ongoing management using herbicides had overall positive outcomes for native plants following successful reduction of invader abundance. This highlights the importance of monitoring beyond the immediate short-term response when evaluating both the effectiveness and biodiversity outcomes of management.

We found effective control of bitou bush was not influenced by fire frequency. Importantly, increased fire frequency did not have any significant negative effects on native vegetation (as can occur if too many fires occur in close succession^38,39^), but was instead associated with higher plant species richness, cover and diversity (Fig. 4). Given bitou bush managed sites are targets for repeated burning, we would have expected to observe reductions in plant species richness with increased fire frequency. Our positive response may indicate either a greater number of fire responding species are facilitated by increased fire than those that are lost, or that fire was applied to sites too infrequently for short-fire intervals to have a detrimental effect. However, increased fire frequency did have a weak negative effect on the abundance of small mammals. The value of using fire as a management approach remains unclear given this negative effect on small mammals, the potential detrimental effects that regular burning can have on native vegetation^38,39^, and our finding that that fire frequency did not significantly effect bitou bush cover.

Existing approaches for generalizing the ecological impact of an invader from many response metrics either ignore the direction of change^5,40^, or categorise impact based solely on the largest effect^4,41^. In either case, important contextual detail is oversimplified regarding whether effects are beneficial or detrimental for the native ecosystem. Bitou bush invasion is generalized as having significant negative impacts on native ecosystems^18,42^. However, we found that while bitou bush cover did negatively impact native plant cover, the abundance of birds and small mammals were benefited. Our results highlight the need to contextually evaluate even well-supported generalisations^43^. Similarly, we demonstrate limitations of simply using the response of the invader to management to make inferences about benefits or success of management. When using the abundance of an invader to indicate other processes (e.g. ecological impact or management effectiveness), we recommend consideration and evaluation of the assumptions underpinning that inference as critical to informing evidence-based management.

## Methods

Animal observation and trapping protocols for this study were approved by the Australian National University Animal Ethics Committee. ﻿All research was conducted in accordance with the applicable institutional, state and national guidelines and regulations for the care of animals.

### Study system and monitoring design

Our study was conducted in Booderee National Park, 200 km south of Sydney, south-eastern Australia (35°10′S, 150°40′E). This temperate region receives average rainfall of approximately 1250 mm per annum spread relatively evenly over the year. The alien perennial shrub, Bitou bush, which is native to South Africa, was intentionally planted to stabilise sand dunes following vegetation clearing and grazing by domestic livestock. This occurred prior to the area being gazetted as a National Park in 1971. Bitou bush subsequently invaded native forest inland of the planted area, and has been subject to ongoing management (of varying effort) since the mid-1990s^44^.

Our study included 54 sites of open forest, located within a ~60 km^2^ area: 46 sites had a *Eucalyptus botryoides* overstorey (>10 m), and eight sites had a *Causuarina glauca* overstorey; all sites had a midstorey of shrubs including *Acacia longifolia* and *Monotoca eliptica* (2–10 m), and an understorey dominated by *Lomandra longifolia* and *Pteridium esculentum* (<2 m)^45,46^. All sites were subject to similar climatic conditions, and had comparable past disturbance histories^44^.

Of the 54 sites in our study, 20 were in forest infested with bitou bush and subject to management (managed sites) and 34 were reference sites where bitou bush had always been absent (control sites). Sites were established in 2007 and set a minimum of 200 m apart (and typically 500 m apart) to ensure spatial dependence in our observations and minimize the risk of management affecting neighbouring sites. The location of dune stabilisation programs (i.e. the primary source of bitou bush invasion) on the Bherwerre Peninsula within the Park meant that uninvaded-control sites tended to be ~500 m further from the coast than invaded sites (see^22^). No sites where bitou bush occurred but remained untreated were included because of a management policy requiring the control of bitou bush in Booderee National Park.

Ongoing bitou bush management in Booderee National Park consists of a combination of herbicide spray and fire treatment, delivered in the sequence spray–fire–spray. First, targeted spraying of ultra-low volume (ULV) glyphosate (15% concentration) by helicopter is undertaken in winter when bitou bush is metabolically active and native plants are relatively inactive^47^. Second, dead bitou bush plants are left to dry for >1 year before being burned by a prescribed fire, typically in winter or early spring. Third, bitou bush seedlings that emerged from fire-cued soil-stored seed are killed by another spray of ULV glyphosate applied approximately 1 year after the fire. No active planting of native vegetation takes place following bitou bush management.

In most cases, the complete spray–fire–spray regime was not applied in full to managed sites due to logistical and resourcing issues with implementing management over several years. Management had also already commenced, at least in part, at all managed sites at the beginning of our study. As such, each managed site has a complex history of full, part or inconsistent management and bitou bush decline or re-establishment. The benefit of this complex legacy is that it has previously enabled the effects of various management sequence combinations on bitou bush cover to be tested, confirming that the spray-fire-spray sequence combination was the most effective in eradicating bitou bush from a site in the short-term^22^. However, the longer-term effects across multiple taxa remain unknown.

### Monitoring biodiversity

Each site contained permanent monitoring plots established along a 100 m transect. Native plants and animals were monitored at all 20 managed sites. Of the 34 control sites, 10 were monitored only for plants, 21 were monitored only for animals, and three were monitored for both. This meant that we did not have suitable controls to directly examine the relationship between total (native + bitou bush) vegetation cover and fauna biodiversity. The difference between reference sites used for plant and animal responses reflects how the design of this study was limited by management priorities and existing long-term biodiversity monitoring programs (see^22,23^). Limited resources also meant that arthropod monitoring could not be included in this study.

Bitou bush was recorded as percent cover of living foliage from four 1 × 1 m plots regularly spaced at 20 m intervals and alternatively offset 20 m from the central transect (from the 20 to 80 m points). All vascular plants within these plots were identified to species and assigned a foliage cover abundance from visual estimate. Multiple small plots per site were used to accurately assess cover and richness responses at a fine scale, and in a consistent and rapid manner in permanent plots through time, rather than examine compositional effects of bitou bush and its management on the vegetation community^22^. Plots were surveyed 15 times from 2007 to 2017, which included four surveys in 2008 (in response to a high amount of management activity at sites), two surveys in 2007, 2009 and 2010, and single surveys in 2011, 2012, 2013, 2015 and 2017.

Birds were surveyed in spring of each year using the ‘point interval count’ method^48^. The numbers of all bird species seen or heard within 5 min and within 50 m of the 20 m and 80 m points of the central transect were recorded. Two surveys were conducted each year during morning hours (~0600–1100) around 2–3 days apart. Bird surveys were undertaken every year from 2007 to 2016 (excluding 2008 when a shortage of staff prevented monitoring).

Reptiles and amphibians (considered collectively as ‘herpetofauna’) were surveyed twice each year (once during spring and once during summer) using permanently placed artificial substrates^49^. Two sheets of corrugated iron (each 1 × 1 m), four standard-sized double roll roofing tiles (42 × 33 cm), and four large wooden railroad sleepers (2.6 m length) were positioned at each of the 20 m and 80 m points along the central transect. Each survey consisted of two site visits on consecutive days, in which substrates were lifted and all reptiles and amphibians present recorded. Herpetofaunal surveys were added to the monitoring program after new resources became available and undertaken every year from 2011 to 2016.

Small mammals were surveyed by trapping at the beginning of summer each year^50^. Three types of trap arrays were used that typically catch the different kinds of small mammal species in our study area (see^46^): (1) 10 Elliot aluminium box traps (10 × 10 × 30 cm) were placed at 10 m intervals along the transect (starting at the 0 m point), four small wire cage traps (20 × 20 × 50 cm) were placed at 20 m intervals along the transect (starting at the 20 m point) and (3) two large wire cage traps (30 × 30 × 60 cm) were place at the 0 m and 100 m points of the transect. Traps were baited with a mixture of peanut butter and rolled oats, and checked daily for three consecutive days. All captured animals were marked with rapidly drying white corrector fluid to ensure re-captured animals were not counted multiple times within a survey. For logistical reasons, only around 65% of uninvaded-control sites are surveyed for small mammals each year (~n = 16). Surveys were undertaken each year from 2007 to 2016.

### Data analysis

Our analysis consisted of two parts that represent the key objectives of management: (1) to effectively reduce the abundance of bitou bush, and (2) to benefit biodiversity by enhancing native species diversity in response to bitou bush control. We addressed these by first testing the effects of ongoing management (considered as frequency of, and time since last, fire and spray application, see Table 1) on live bitou bush cover. We then assessed the response of biodiversity indicators (richness, abundance and diversity) to both the effects of ongoing management and the amount of live bitou bush present post management (Table 2).

**Table 2:** Variables used in analysis.

Variable	Description	Range	Type
***Fixed effects***			
Fire frequency	Total number of fires recorded as occurring at a site between 1964 and the year of an observation.	0 to 4 (median = 1)	Integer
Time since last fire	Time in years (rounded to whole number) since the most recent fire event.	0 to 33 (median = 8)	Integer
Spray frequency	Total number of sprays recorded as occurring at a site between 1964 and the year of an observation.	0 to 7 (median = 2)	Integer
Time since last spray	Time in years (rounded to whole number) since the most recent spray event.	0 to 12 (median = 2)	Integer
Live bitou bush cover (%)	Average cover of live bitou bush per site	0 to 77	Integer
***Random effects***			
“Site”: to account for non-independence from repeated-measures	Site identifier code unique to all 54 monitoring sites included in this study.	—	Factor (54 levels)
“Site location”: to account for spatial auto-correlation	Broad classification based on whether site is within the zone invaded by bitou bush and subject to management, or not.	Inside Outside	Factor (2 levels)
“Survey year”: to account of temporal auto-correlation	Year in which monitoring was undertaken (2007–2017).	1 to 10	Integer

#### Bitou bush response to management

Using data from only the managed sites, we fitted generalised linear mixed models^51^ using proportional bitou bush foliage cover as the response variable, with binomial error distributions and a logit-link function. These data were mean values for each site (from four plots), for each of the 15 vegetation survey periods over 10 years (n = 283 observations). Bitou bush cover was modelled as a function of fire frequency and the interaction of spray frequency and time since spray (full model). We did not include time since fire in the regression model due to a moderately strong correlation with fire frequency (r = 0.68). All predictor variables were scaled to a mean of zero and a standard deviation of one prior to modelling to allow direct comparison of regression coefficients. We used Akaike’s Information Criterion corrected for small sample sizes (AICc) to rank subsets of the full model^52^. Plots of residuals against fitted values, residual frequency histograms, quantile-quantile plots and residual variation box plots were examined to verify homogeneity and expected properties of residuals^53^. Tests for overdispersion were undertaken to assess whether there was additional variance in the data than assumed by the Poisson or negative binomial distributions^54^. We used mixed models to account for non-independence resulting from repeated-measures with the inclusion of a site-level random effect for all models.

All analyses were performed using R version 3.5.0^55^. Models were fitted using the ‘glmer’ function in the “lme4” package^56^, subsets of the full model were ranked using the ‘dredge’ function in the “MuMIn” package^57^, and coefficients and 95% confidence intervals for coefficients of the top-ranked models (lowest AICc) were estimated using the ‘confint’ function in the “stats” package.

#### Biodiversity response to bitou bush re-establishment and management

Using data from both the managed and control sites, we analysed species richness, abundance (average cover for plants, number of individuals for fauna), and diversity (Shannon’s Diversity Index) in four datasets (plants, birds, herpetofauna and small mammals) in response to 10-years of bitou bush invasion and management intervention. All datasets comprised repeat visits to 44 permanent monitoring sites and our units of analysis were site-level surveys for plants (pooled 4 plots per site, n = 412 observations), birds (pooled four point counts per site, n = 388), herpetofauna (pooled four searches per site, n = 449), and small mammals (pooled three trap nights per site, n = 431). We did not fit models for herpetofauna diversity as most of our observations (80%, n = 360) were of one or zero species (i.e. no diversity).

We used the same approach of fitting generalised linear mixed models, determining the top-ranked model, testing for fit and overdispersion, and making model predictions for our biodiversity response variables as we did for the response of bitou bush to management. Biodiversity indicators were modelled as a function of bitou bush cover, fire frequency, time since spray and all pairwise interactions of those variables (full model) (see Table 2). Time since fire was excluded as in the previous analysis, with spray frequency also excluded due to a strong correlation with live bitou bush cover (r = −0.72). Along with a site-level random effect, we included ‘area’ (inside vs outside the bitou bush infested area) and survey year in all models to account for spatial and temporal autocorrelation in the data respectively^58^ (Table 2). Similarly, an additional ‘season’ random effect was included in herpetofauna response models to account for seasonal variation in reptile activity. Continuous predictor variables were standardised, subsets of the full models ranked, and the top-ranked model used to make predictions as in the first analysis. If the top-ranked model did not include bitou bush cover, but bitou bush cover did feature in a model considered to be well-supported (ΔAICc < 2), predictions were made using the latter. We modelled species richness and animal abundance response variables with Poisson distributions and log-link functions. Native plant cover (%) and Shannon Diversity Indices response variables were modelled with negative binomial distributions and logit-link functions.

## Supplementary information

Supplementary information

## Data Availability

The datasets generated and analyzed during the current study are part of the Long-Term Ecological Research Network (LTERN) in Australia and are available at the LTERN Data Portal (www.ltern.org.au/knb/). Data packages used in this study: Plot details (ltern7.73.22), Plants (ltern2.291.63), Birds (ltern7.30.37), Herpetofauna (ltern7.110.22) and Small mammals (ltern7.121.19).
