# Supplementary material for: Invasive shrub re-establishment following management has contrasting effects on biodiversity
Source: Sci Rep. 2019 Mar 11;9:4083. doi: 10.1038/s41598-019-40654-y (PMC6412044; doi:10.1038/s41598-019-40654-y)
Supplement: Supplementary file 1 — Supplementary information [file 41598_2019_40654_MOESM1_ESM.pdf]

Supplementary information

Invasive shrub re-establishment following management has contrasting effects on biodiversity

Luke S. O'Loughlin[1,2*], Ben Gooden[3,4], Claire N. Foster[1], Christopher I. MacGregor[1,5], and Jane A. Catford[6,7] and David B. Lindenmayer[1,5]

[1]Fenner School of Environment and Society, The Australian National University, Canberra, ACT 2601, Australia

[2]School of Environmental Science, Charles Sturt University, Albury, NSW 2640, Australia

[3]Centre for Sustainable Ecosystem Solutions, School of Biological Sciences, University of Wollongong, Wollongong, NSW 2522, Australia

[4]CSIRO Health and Biosecurity, GPO Box 1700, Canberra, ACT 2601, Australia

[5]Threatened Species Recovery Hub of the National Environment Science Programme, Fenner School of Environment and Society, The Australian National University, Canberra, ACT 2601, Australia

[6]Biological Sciences, University of Southampton, Southampton, SO17 1BJ, UK

[7]Department of Geography, King's College London, London, WC2B 4BG, UK

[*] luke.oloughlin@anu.edu.au

Table S1. Combined species list of all observed plants, birds, herpetofauna and mammals over the 10-years of our study. Species are ranked from most to least common as calculated by either % of total quadrats the species was recorded in (plants) or % contribution to the total number of observed individuals (birds, herpetofauna and mammals). ***denotes non-native species. [R]denotes reptile. [A]denotes amphibian.

Species	%	Species	%
**Plants**			
Imperata cylindrica var. *major*	60.6	*Marsdenia suaveolens*	0.6
Pteridium esculentum	52.2	*Poa* sp.	0.6
Lomandra longifolia	35.5	*Desmodium rhytidophyllum*	0.5
Desmodium varians	31.3	*Hardenbergia violacea*	0.5
Oplismenus aemulus	29.5	*Leptospermum polygalifolium*	0.5
Microlaena stipoides var. *stipoides*	28.0	*Senecio* sp.	0.5
*Chrysanthemoides monilifera**	27.5	*Acacia ulicifolia*	0.5
Entolasia marginata	25.0	*Bossiaea ensata*	0.5
Schelhammera undulata	20.0	*Lomandra filiformis* subsp. *filiformis*	0.5
Stephania japonica var. *discolor*	18.1	*Pittosporum revolutum*	0.5
Parsonsia straminea	17.2	*Carex appressa*	0.4
Dichondra repens	16.2	*Desmodium brachypodum*	0.4
Geranium homeanum	16.2	*Galium gaudichaudii*	0.4
Glycine tabacina	15.8	*Smilax glyciphylla*	0.4
Marsdenia rostrata	15.6	*Sporobolus virginicus*	0.4
Monotoca elliptica	14.6	*Corybas* sp.	0.4
Hibbertia scandens	13.8	*Eucalyptus botryoides*	0.4
Commelina cyanea	13.7	*Lomandra multiflora* subsp. *Multiflora*	0.4
Gonocarpus teucrioides	13.0	*Plantago debilis*	0.4
Cyperus gracilis	12.0	*Cyperus polystachyos*	0.3
Dianella caerulea	11.4	*Eragrostis brownii*	0.3
Viola hederacea	9.3	*Astroloma humifusum*	0.2
Oplismenus imbecillis	9.0	*Einadia hastata*	0.2
Casuarina glauca	8.6	*Hypochaeris radicata**	0.2
Acianthus fornicatus	8.3	*Juncus continuus*	0.2
Poranthera microphylla	8.1	*Leptospermum trinervium*	0.2
Glycine clandestina	8.0	*Lomandra* sp.	0.2
*Hydrocotyle bonariensis**	7.1	*Myoporum boninense* subsp. *australe*	0.2
Pittosporum undulatum	6.7	*Patersonia sericea*	0.2
Baumea juncea	6.0	*Smilax australis*	0.2
Banksia integrifolia	5.6	*Astroloma pinifolium*	0.2
Lepidosperma concavum	5.4	*Caladenia carnea*	0.2
Eucalyptus sp.	5.3	*Galium australe*	0.2
Acacia longifolia	5.2	*Hibbertia* sp.	0.2
Breynia oblongifolia	4.8	*Histiopteris incisa*	0.2
Veronica calycina	4.5	*Solanum* sp.	0.2
Elaeocarpus reticulatus	3.4	*Synoum glandulosum*	0.2
Senecio minimus	3.4	*Caladenia alata*	0.1
Lagenifera stipitata	3.1	*Caladenia picta*	0.1
Oxalis sp.	3.1	*Casuarina* sp.	0.1
Hibbertia linearis	3.0	*Centaurium tenuiflorum**	0.1
Hydrocotyle peduncularis	3.0	*Cissus hypoglauca*	0.1
Banksia serrata	2.9	*Claoxylon australe*	0.1
Pratia purpurascens	2.9	*Corybas pruinosus*	0.1
Echinopogon ovatus	2.7	*Goodenia heterophylla* subsp. *eglandulosa*	0.1
Eustrephus latifolius	2.7	*Hypolepis muelleri*	0.1
Themeda australis	2.5	*Morinda jasminoides*	0.1
Pterostylis sp.	2.4	*Notelaea ovata*	0.1
Solanum stelligerum	2.4	*Orchid* sp.	0.1
Leucopogon lanceolatus	2.2	*Persoonia linearis*	0.1
Notelaea longifolia forma *longifolia*	2.0	*Poranthera ericifolia*	0.1

Oxalis perennans	1.8	*Pterostylis concinna*	0.1
Entolasia stricta	1.8	*Senecio bipinnatisectus*	0.1
Eucalyptus pilularis	1.7	*Solanum prinophyllum*	0.1
Galium propinquum	1.7	*Anisopogon avenaceus*	0.1
Acianthus sp.	1.7	*Austrostipa mollis*	0.1
Ficinia nodusa	1.7	*Banksia* sp.	0.1
Asteraceae sp.	1.6	*Baumea acuta*	0.1
Bossiaea heterophylla	1.6	*Caesia parviflora*	0.1
*Oxalis articulata**	1.6	*Carthamus lanatus*	0.1
Kennedia rubicunda	1.5	*Corymbia gummifera*	0.1
Coronidium elatum	1.5	*Cyperus congestus*	0.1
*Anagallis arvensis**	1.4	*Epacris* sp.	0.1
Unidentified seedling	1.4	*Epaltes australis*	0.1
*Conyza bonariensis**	1.3	*Euchiton gymnocephalus*	0.1
Poa labillardieri	1.3	*Glochidion ferdinandi*	0.1
Tetratheca thymifolia	1.3	*Glycine microphylla*	0.1
*Conyza parva**	1.2	*Gomphocarpus fruticosus*	0.1
Corybas fimbriatus	1.2	*Gonocarpus micranthus subsp_ micranthus*	0.1
Cynodon dactylon	1.2	*Goodenia* sp.	0.1
Zoysia macrantha	1.2	*Hibbertia aspera*	0.1
Clematis aristata	1.1	*Isolepis* sp.	0.1
Gahnia clarkei	1.1	*Lachnagrostis filiformis*	0.1
Acacia implexa	1.0	*Lomandra cylindrica*	0.1
Calochlaena dubia	1.0	*Lomandra glauca*	0.1
Solanum pungetium	1.0	*Opercularia aspera*	0.1
Billardiera scandens	0.8	*Opercularia hispida*	0.1
*Oxalis corniculate**	0.8	*Poranthera corymbosa*	0.1
Caladenia catenata	0.8	*Pseudognaphalium luteoalbum*	0.1
Gnaphalium involucratum	0.8	*Pterostylis pedunculata*	0.1
Kennedia prostrata	0.7	*Schizaea bifida*	0.1
Leptospermum laevigatum	0.7	*Senecio linearifolius*	0.1
Platysace lanceolata	0.7	*Styphelia triflora*	0.1
Leucopogon parviflorus	0.7	*Trema aspera*	0.1
*Solanum nigrum**	0.7	Unidentified grass	0.1
Acacia longissima	0.6	*Urtica incisa*	0.1
Acacia sp.	0.6	*Zieria smithii*	0.1
**Birds**			
Yellow-faced Honeyeater	12.0	Black-faced Monarch	0.1
Rainbow Lorikeet	7.1	Masked Lapwing	0.1
Grey Fantail	5.5	Eastern Koel	0.1
White-throated Treecreeper	4.3	Brown Goshawk	0.1
Brown Thornbill	4.3	Galah	0.1
Spotted Pardalote	4.2	Grey Goshawk	<0.1
Eastern Spinebill	3.9	Striated Pardalote	<0.1
Red Wattlebird	3.9	Welcome Swallow	<0.1
Silvereye	3.8	Australian Pelican	<0.1
Eastern Whipbird	3.7	Bassian Thrush	<0.1
Noisy Friarbird	3.5	White-eared Honeyeater	<0.1
White-browed Scrubwren	3.3	Red-browed Treecreeper	<0.1
Lewin's Honeyeater	2.8	Rufous Fantail	<0.1
Crimson Rosella	2.8	Wonga Pigeon	<0.1
Little Wattlebird	2.7	Australian Magpie	<0.1
Striated Thornbill	2.7	Glossy Black-Cockatoo	<0.1
New Holland Honeyeater	2.4	Pacific Black Duck	<0.1
Golden Whistler	2.3	White-faced Heron	<0.1
Grey Shrike-thrush	2.2	Channel-billed Cuckoo	<0.1
Variegated Fairy-wren	1.8	Common Bronzewing	<0.1
Eastern Yellow Robin	1.7	Magpie-lark	<0.1
Olive-backed Oriole	1.6	Rose Robin	<0.1
Fan-tailed Cuckoo	1.6	Wedge-tailed Eagle	<0.1

Rufous Whistler	1.5	Australian Pied Oystercatcher	<0.1
Brown Gerygone	1.3	Brush Bronzewing	<0.1
Laughing Kookaburra	1.0	Crescent Honeyeater	<0.1
Scarlet Honeyeater	0.9	Fuscous Honeyeater	<0.1
Pied Currawong	0.9	Large-billed Scrubwren	<0.1
Superb Fairy-wren	0.9	Silver Gull	<0.1
Shining Bronze-Cuckoo	0.9	Collared Sparrowhawk	<0.1
White-naped Honeyeater	0.7	Little Pied Cormorant	<0.1
Satin Bowerbird	0.7	Sulphur-crested Cockatoo	<0.1
Grey Butcherbird	0.6	Black Swan	<0.1
Eastern Bristlebird	0.6	Dollarbird	<0.1
Australian King-Parrot	0.6	Eastern Rosella	<0.1
Australian Raven	0.5	Mistletoebird	<0.1
Little Lorikeet	0.5	Peaceful Dove	<0.1
White-cheeked Honeyeater	0.5	Pheasant Coucal	<0.1
Black-faced Cuckoo-shrike	0.4	Pied Cormorant	<0.1
Yellow-tailed Black-Cockatoo	0.4	Sooty Oystercatcher	<0.1
Red-browed Finch	0.3	Spectacled Monarch	<0.1
Brown-headed Honeyeater	0.3	Swift Parrot	<0.1
Leaden Flycatcher	0.3	Tawny-crowned Honeyeater	<0.1
Yellow Thornbill	0.3	Australasian Darter	<0.1
Musk Lorikeet	0.2	Australian Hobby	<0.1
Gang-gang Cockatoo	0.2	Australian White Ibis	<0.1
Horsfield's Bronze-Cuckoo	0.2	Bar-shouldered Dove	<0.1
Sacred Kingfisher	0.2	Brown Falcon	<0.1
Crested Shrike-tit	0.1	Eastern Great Egret	<0.1
Topknot Pigeon	0.1	Pallid Cuckoo	<0.1
Varied Sittella	0.1	Peregrine Falcon	<0.1
Little Black Cormorant	0.1	Southern Boobook	<0.1
Whistling Kite	0.1	Superb Lyrebird	<0.1
White-bellied Sea-Eagle	0.1	White-bellied Cuckoo-shrike	<0.1
**Herpetofauna**			
Lampropholis delicata[R]	64.0	*Litoria jervisiensis*[A]	0.5
Cryptophis nigrescens[R]	19.6	*Limnodynastes tasmaniensis*[A]	0.3
Crinia signifera[A]	5.5	*Cyclodomorphus michaeli*[R]	0.2
Pseudophryne bibronii[A]	2.3	*Hemiaspis signata*[R]	0.2
Lampropholis guichenoti[R]	1.7	*Paracrinia haswelli*[A]	0.2
Pseudechis porphyriacus[R]	1.5	*Amphibolurus muricatus*[R]	0.1
Limnodynastes peronii[A]	1.3	*Limnodynastes dumerilii*[A]	0.1
Acritoscincus platynotus[R]	1.0	*Pseudonaja textilis*[R]	0.1
Uperoleia tyleri[A]	0.7	*Tiliqua scincoides*[R]	0.1
Eulamprus quoyii[R]	0.5		
**Mammals**			
Antechinus stuartii	47.7	*Tachyglossus aculeatus*	0.4
Rattus fuscipes	34.1	*Antechinus swainsonii*	0.2
Trichosurus vulpecula	10.6	*Rattus rattus**	0.2
Perameles nasuta	4.3	*Oryctolagus cuniculus**	0.1
Rattus lutreolus	1.1	*Macropus rufogriseus*	<0.1
Cercartetus nanus	0.7	*Pseudocheirus peregrinus*	<0.1
Wallabia bicolor	0.4		

Table S2. Model selection tables for the generalized linear mixed models (GLMMs) testing the relationship between properties of ongoing management (SF: spray frequency, FF: fire frequency, TSLS: time since last spray) and bitou bush re-establishment (**A**), and the relationship between bitou bush re-establishment (BB) and on-going management and biodiversity response variables (**B**–**L**). Details are provided for the top four models along with the null model.

A. Bitou bush cover

Model rank	Model terms				K	LL	AICc	ΔAICc	AICcWt
	SF	FF	TSLS	SF : FF					
1	+				3	-15.1	36.1	0.00	0.260
2	+	+			4	-14.5	37.0	0.87	0.168
3	+		+		4	-14.8	37.6	1.44	0.126
4	+	+		+	5	-13.8	37.6	1.45	0.125
null					2	-19.0	41.9	5.77	0.014

B. Plant species richness

Model rank	Model terms			K	LL	AICc	ΔAICc	AICcWt
	BB	FF	BB : FF					
1	+	+	+	7	-1138.5	2290.6	0.00	0.363
2		+		5	-1140.4	2291.0	0.40	0.298
3	+	+		6	-1139.4	2291.1	0.48	0.286
4	+			5	-1142.8	2295.8	5.12	0.028
null				4	-1143.9	2296.0	5.34	0.025

C. Bird species richness

Model rank	Model terms			K	LL	AICc	ΔAICc	AICcWt
	BB	FF	BB : FF					
1	+			5	-1177.6	2365.4	0.00	0.425
null				4	-1179.2	2366.5	1.13	0.241
3	+	+		6	-1177.6	2367.4	1.98	0.158
4		+		5	-1179.1	2368.4	3.05	0.092
5	+	+	+	7	-1177.2	2368.6	3.25	0.084

D. Herpetofauna species richness

Model rank	Model terms			K	LL	AICc	ΔAICc	AICcWt
	BB	FF	BB : FF					
1		+		6	-523.0	1058.2	0.00	0.427
null				5	-524.5	1059.1	0.96	0.263
3	+	+		7	-523.0	1060.2	2.02	0.155
4	+			6	-524.5	1061.2	3.01	0.095
5	+	+	+	8	-522.9	1062.1	3.93	0.060

E. Small mammal species richness

Model rank	Model terms			K	LL	AICc	ΔAICc	AICcWt
	BB	FF	BB : FF					
1		+		4	-642.2	1295.4	0.00	0.426
2	+	+		5	-642.7	1296.4	1.01	0.258
null				6	-644.9	1297.8	2.43	0.128
4	+	+	+	5	-642.1	1298.2	2.81	0.105
5	+			7	-644.3	1298.7	3.38	0.081

F. Plant cover

Model rank	Model terms					K	LL	AICc	ΔAICc	AICcWt
	BB	FF	TSLS	BB : FF	BB : TSLS					
1	+	+	+			7	-176.6	367.1	0.00	0.417
2	+	+	+	+		8	-176.1	368.2	1.06	0.245
3	+	+	+		+	8	-176.3	368.7	1.53	0.194
4	+	+	+	+	+	9	-175.8	369.5	2.38	0.126
null						4	-239.3	486.5	119.39	<0.001

G. Bird abundance

Model rank	Model terms			K	LL	AICc	ΔAICc	AICcWt
	BB	FF	BB : FF					
1	+			6	-1914.9	3842.1	0.00	0.474
2	+	+	+	8	-1913.5	3843.4	1.24	0.252
3	+	+		7	-1914.8	3842.8	1.71	0.201
null				5	-1918.2	3846.6	4.51	0.050
5		+		6	-1918.0	3848.3	6.18	0.022

H. Herpetofauna abundance

Model rank	Model terms			K	LL	AICc	ΔAICc	AICcWt
	BB	FF	BB : FF					
null				5	-925.7	1861.6	0.00	0.431
2		+		6	-925.3	1862.8	1.24	0.232
3	+			6	-925.5	1863.1	1.58	0.196
4	+	+		7	-925.1	1864.4	2.86	0.103
5	+	+	+	8	-925.1	1866.4	4.87	0.038

I. Small mammal abundance

Model rank	Model terms					K	LL	AICc	ΔAICc	AICcWt
	BB	FF	TSLS	BB : FF	BB : TSLS					
1	+	+	+			7	-745.3	1504.6	0.00	0.493
2	+	+	+	+		8	-745.2	1506.5	1.88	0.193
3	+	+	+		+	8	-745.3	1506.6	1.99	0.183
4	+	+	+	+	+	9	-745.2	1508.5	3.88	0.070
null						4	-1288.4	2584.8	1080.19	<0.001

J. Plant diversity

Model rank	Model terms			K	LL	AICc	ΔAICc	AICcWt
	BB	FF	BB : FF					
1		+		6	-135.5	283.1	0.00	0.945
null				5	-139.8	289.7	6.62	0.034
3	+	+		7	-138.3	290.9	7.81	0.019
4	+			6	-142.5	297.2	14.14	0.001
5	+	+	+	8	-141.2	298.7	15.58	<0.001

K. Bird diversity

Model rank	Model terms			K	LL	AICc	ΔAICc	AICcWt
	BB	FF	BB : FF					
null				5	-89.3	188.8	0.00	0.960
2		+		6	-92.1	196.4	7.59	0.022
3	+			6	-92.3	196.8	7.97	0.018
4	+	+		7	-95.1	204.4	15.59	<0.001
5	+	+	+	8	-97.6	211.6	22.82	<0.001

L. Small mammal diversity

Model rank	Model terms				K	LL	AICc	ΔAICc	AICcWt
	BB	FF	TSLS	BB : TSLS					
1			+		6	37.2	-62.4	0.00	0.404
2		+	+		7	37.2	-60.5	1.95	0.152
3	+		+		7	-37.2	-60.4	1.98	0.150
4	+		+	+	8	-37.7	-59.4	3.06	0.087
null					4	-34.0	-57.9	4.50	0.042
